# Dentistry a Distinct and Independent Profession

**Published:** 1896-05

**Authors:** R. Finley Hunt

**Affiliations:** Washington, D. C.


ARTICLE VIII.

### DENTISTRY A DISTINCT AND INDEPENDENT PROFESSION.

BY R. FINLEY HUNT, D. D. S., WASHINGTON, D. C.

A POSITION BASED UPON UNDENIABLE HISTORICAL AND CURRENT FACTS, AND NOT UPON THEORIES SUPPORTED BX ARGUMENTS.

Read before the Washington City Dental Society at its 29th Annual Meeting. Unanimously Adopted and Ordered to be Printed.

From the newspaper reports of the decision of Judge Miller, October 31st, in the case of Dr. Burke, charged with violation of the District dental law, there seemed to be a great misapprehension on the part of the counsel for the defense as to the difference in the character and powers of colleges and universities, as well as to the standing of dentistry among the learned professions and its relation to the medical profession. This want of information seems to be general among the members of all professions and the people of all classes. The object of the writer of this paper is to supply as well as he can this information, and in doing so he will consider colleges and universities as literary institutions established and intended to instruct students in the various branches of learning required for their chosen professions or callings and, when they prove themselves properly qualified, to attest their proficiency therein by diplomas or certificates, and when so provided to confer upon them the distinctive degree to which they are entitled.

Judge Miller in his decision referred to the terms of the charter of the National Homeopathic Medical College and said, "that under these terms he thought the college could not give a degree in dentistry. The charter mentioned nothing of a chair of dentistry." That is to say this Medical College was chartered, as all medical colleges are, to give a medical education to its students and to confer the degree of Doctor of Medicine on such of them as are qualified to receive it. So a Dental college is chartered to give a dental education and to confer the degree of Doctor of Dental Surgery or its equivalent on its graduates. These and all similar institutions are limited in their power of certification of qualification of their graduates and cannot properly assume to confer upon them the degree belonging to another profession.

On the other hand a university, as its name implies, is a universal school in which are taught all branches of learning or the four faculties of theology, medicine, law, and the sciences and arts; indeed it is an assembly of colleges, established in any place with professors for instructing students in the sciences and other branches of learning and where degrees are conferred.

There are about six universities in the District of Columbia, some of which have been in operation for a number of years, while others have not yet been fully organized. Three of these have, each of them within a few years past, established in addition to their other departments or colleges, a department or college of dentistry, which has its own faculty, its own curriculum and whose graduates receive the special and distinctive degree of Doctor of Dental Surgery, a degree that is independent of and not connected with any other one. There is a number of universities in the States that have dental departments or colleges, in all which, so far as the writer of this knows, the same state of facts exist.

These facts undoubtedly give to dentistry, so far as the universities are concerned, a position among the learned professions and place it on the same plane as the other learned professions acquired in the universities.

Prior to the period of their establishment in universities, dental colleges had been chartered by different States and empowered to give instruction and confer degrees in dentistry, the oldest one, the Baltimore College of Dental Surgery, dating back to 1839. This fact and date mark the foundation and beginning of the conversion into a profession of what had before been simply a vocation or calling, or, we may say, the beginning of the birth of a new profession which at this time has all the elements and characteristics necessary to constitute it such. Since that date others have been chartered (some in connection with medical colleges) and now in 1895 there are forty-eight dental colleges in the United States. Thirty-eight of these are pledged, through membership in the National Association of Dental Faculties, to allow no one to matriculate without first giving proof by examination or otherwise of having received at least a good English education and to exact from every student in attendance on three full courses of lectures and clinical instruction before examination for graduation. The other ten will doubtless soon follow and give the same pledge, as that step is necessary before they can have a recognized standing as proper dental educational institutions. That this pledge will be kept is assured because any dental college failing to do so will forfeit its standing in the profession.

The members of the dental profession of the United States have been engaged for some years past in the effort to elevate the standard of dental education by improving and unifying the requirements and curriculum of all the dental colleges in the country. As one means to this end two bodies have been formed, the National Association of Dental Faculties and the National Association of Dental Examiners, composed respectively of delegates from dental faculties and from State boards of dental examiners, and meeting annually. They have already done much good and are steadily accomplishing the object of their organization.

The American Medical Association at the meeting in

Chicago adopted, June 10th, 1887, the following resolution:

"*Resolved*, that the regular graduates of such dental and oral schools and colleges as require of their students a standard of preliminary or general education and a term of professional study equal to the best class of the medical colleges of this country and embrace in their curriculum all the fundamental branches of medicine, differing chiefly by substituting practical and clinical instruction in dental and oral medicine and surgery in place of practical and clinical instruction in general medicine and surgery, be recognized as members of the regular profession of medicine and eligible to membership in this association, on the same conditions and subject to the same regulations as other members."

There has been no occasion, so far as the writer of this knows, for the American Medical Association to construe in scope and detail the intention and meaning of this resolution, so that we must be guided in our understanding of it by its language and the circumstances under which it was adopted. It would require too much space to detail the circumstances existing at the time. It will be sufficient to say that there was then a crisis which seemed to call for some such action. The American Medical Association gracefully met the crisis by voluntarily adopting the above liberal resolution.

The resolution conceded that the education acquired in a dental school or college conducted as most of them were at that time and are now, was such as to entitle its graduates to honorary membership in the regular medical profession and to eligibility to active membership in the American Medical Association. That association could not confer upon dental graduates the degree of M. D., nor could it authorize them to practice medicine. Its action was then plainly an acknowledgement that the education acquired in certain schools and colleges, other than medical ones, was as much of a professional one as that acquired in medical colleges, and therefore constituted the graduates of such schools and colleges members of a distinct and independent profession, whom by virtue of such education and membership it admitted to honorary membership in the regular medical profession and declared eligible to membership in its organization. This seems to me to be the only proper construction and effect of the resolution, which reflects credit on both professions,—the older one for its liberality and the younger one for its attainments. In the same year dentists were, by virtue of the degree they hold from dental colleges, admitted to membership in the International Congress at its meeting in Washington City.

In the District of Columbia practically, and in many of the States by enactment, members of the dental profession are exempted from jury duty, as are those of the medical and legal professions.

Congress and nearly all the State legislatures have enacted dental laws requiring a thorough dental education on the part of any person desiring to commence the practice of dentistry, and have provided for the appointment; in each of their several jurisdictions, of a Board of Dental Examiners, whose duty it is to see that the applicants for license to practice dentistry possess the requisit education and whose approval and certificate to that effect are necessary before such applicants are allowed to practice.

From this statement of facts, historical and current, it will be seen that dentistry is, by competent authorities, acknowledged and regarded as a profession equal to and independent of all others, and that its qualifications, acquirements and equipments,—scientific, artistic and organic,—entitle it to the fourth place in the family of the learned professions, the other three being those of Medicine, Law and Theology.

